# Supplementary material for: DeepCycle reconstructs a cyclic cell cycle trajectory from unsegmented cell images using convolutional neural networks
Source: Mol Syst Biol. 2020 Oct 6;16(10):e9474. doi: 10.15252/msb.20209474 (PMC7537830; doi:10.15252/msb.20209474)
Supplement: Supplementary file 1 — Appendix [file MSB-16-e9474-s001.pdf]

APPENDIX.

DeepCycle reconstructs a cyclic cell cycle trajectory from unsegmented cell images using convolutional neural networks

Table of contents

Appendix Figure S1……………………………………………………………………………………2

Appendix Figure S2………………………………………………………………………………....3

Appendix Figure S3…………………………………………………………………………………4-5

Appendix Figure S4……………………………………………………………………………………5

Appendix Figure S5……………………………………………………………………………………6

Appendix Figure S6……………………………………………………………………………………7

Appendix Figure S7……………………………………………………………………………………8

Appendix Figure S8……………………………………………………………………………………9

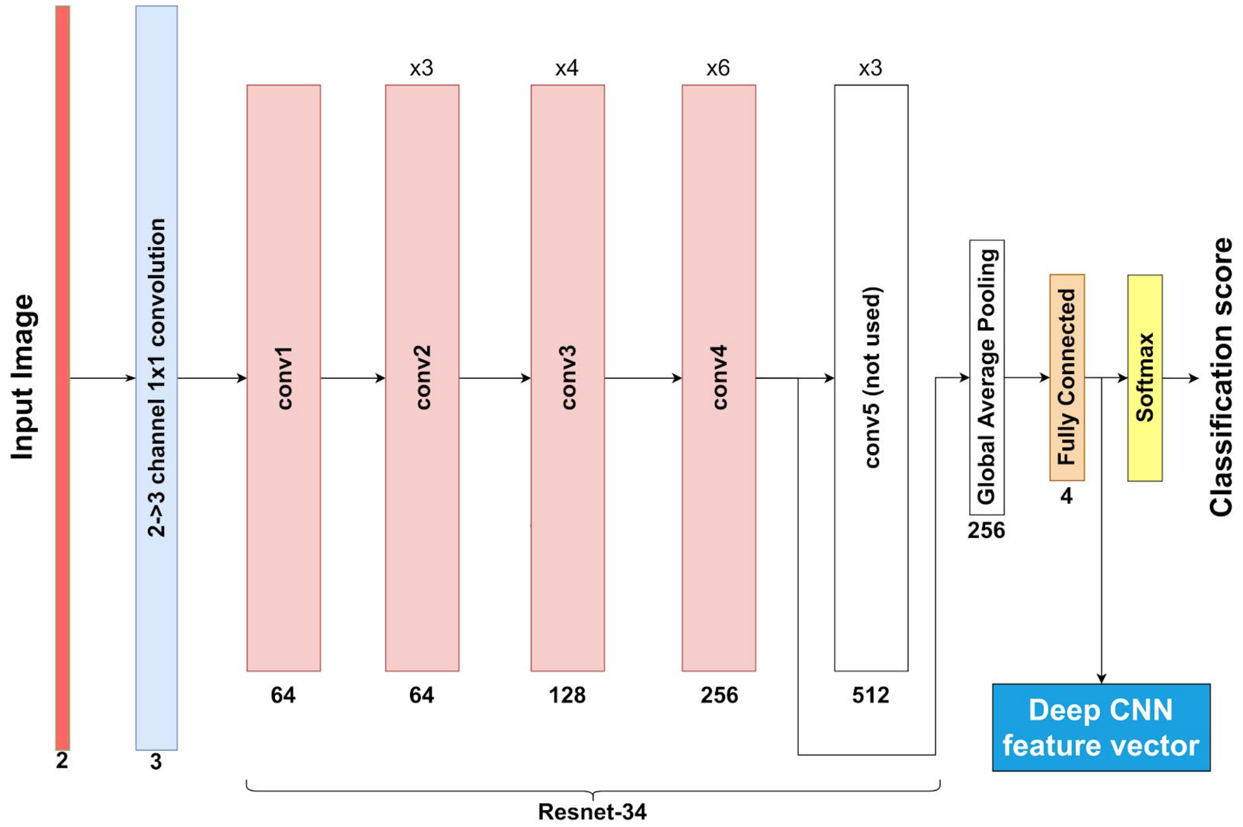

Appendix Figure S1: Architecture of the DeepCycle neural net. A 2-channel cell image is transformed into a 3-channel image using a 1x1 convolutional layer and fed to Resnet-34 pre-trained on Imagenet. Intermediate Conv4 activations, after average pooling, are fed to a fully connected layer and Softmax. Conv5 block is not used. Softmax generates the probabilities of a cell to belong to the virtual classes. The output of the fully connected layer represents a 4-component cell feature vector used in UMAP visualization.

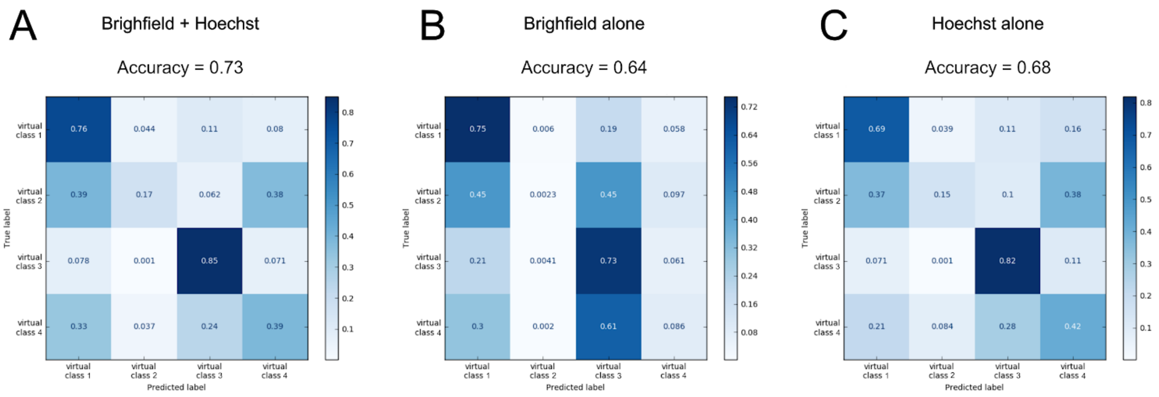

Appendix Figure S2: Virtual class classification accuracies and confusion matrices of the DeepCycle network classifier. During the training phase of the DeepCycle network, dual channel cell images are classified into the four virtual classes defined on the FUCCI2 fluorescence intensity plane (mAG-Geminin and mKO2-Cdt1). We report the classification accuracies as well as the confusion matrices for each class with the DeepCycle network trained from cell images with both the brightfield and hoechst channels as input (A), brightfield alone (B) and Hoechst alone (C). The confusion matrices and classification accuracies were calculated on test data.

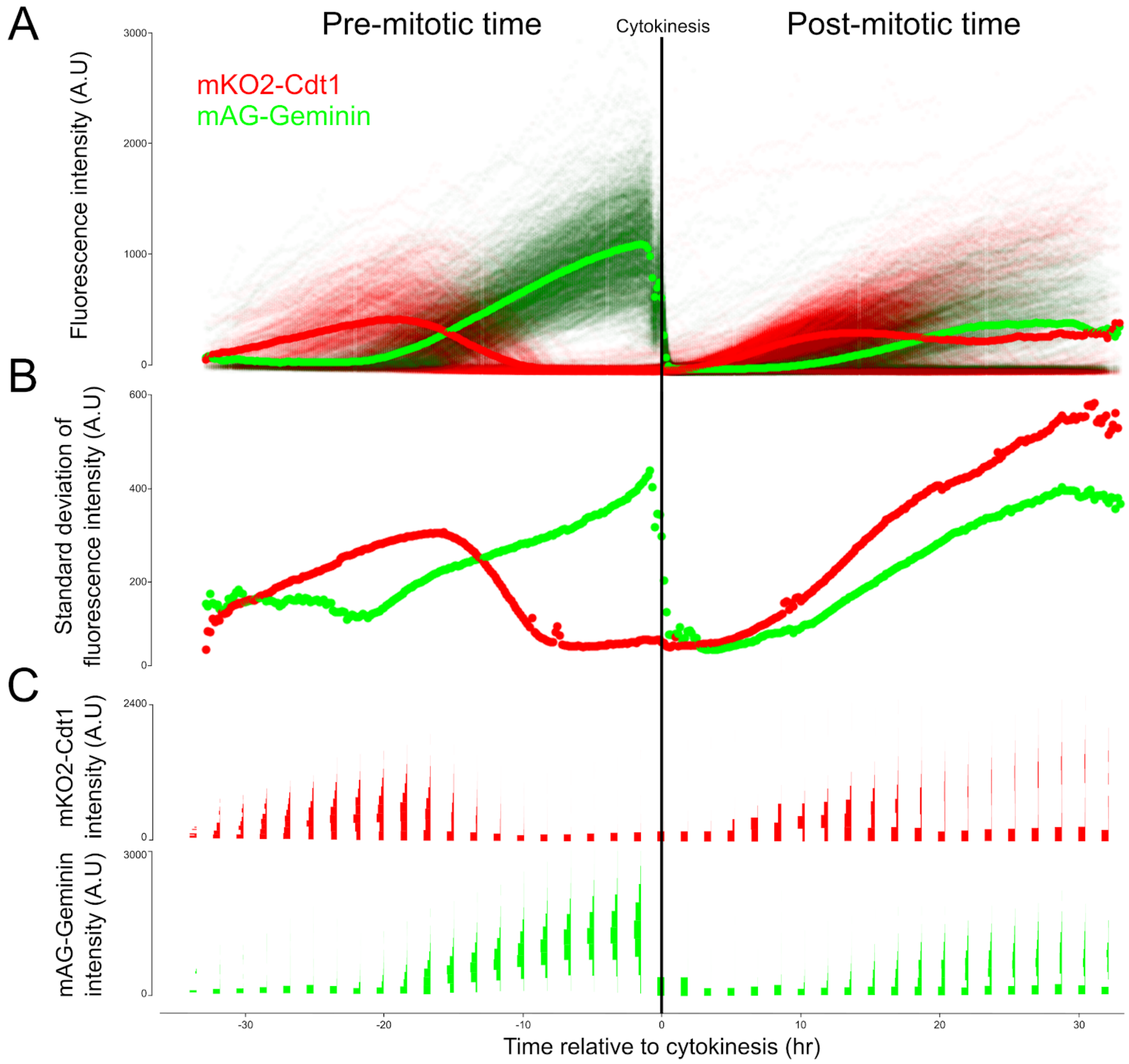

Appendix Figure S3: **FUCCI2 fluorescence signal heterogeneity among pre- and post-mitotic times**. **A**. Fluorescence intensities of the FUCCI2 mAG-Geminin and mKO2-Cdt1 reporters for 1024 tracks as a function of the time relative to the division event (cytokinesis). Each track has been manually validated and the division frame has been labeled. The X coordinate value is the time of measurement subtracted by the time of division. The fluorescence intensities are the mean pixel values over a square of 13.2x13.2 μm centered around the centroid of the cell nucleus in the GFP or the Cy3 channels for the mAG-Geminin or the mKO2-Cdt1 readout, respectively. Typically, all cells measured before the division event (negative time values) are committed to divide and present the typical FUCCI2 cell cycle trends. On the other hand, not every cell which has already divided (positive time values) will divide again, introducing more heterogeneity in the FUCCI2 fluorescence readout. **B.** Quantification of the FUCCI2 readout heterogeneity for the pre- and post-mitotic times. The pre-mitotic median standard deviation of the FUCCI2 intensities are lower than the post-mitotic intensities (median

standard deviation of pre- and post-mitotic times are 187.9, 340 for mKO2-Cdt1 ***; 216.0, 225.6 for mAG-Geminin N.S.). **C.** Distribution of mKO2-Cdt1 intensities (red) and mAG-Geminin (green). The cell distributions are unimodal for the cells in their pre-mitotic times. A bimodal trend appears in both the mKO2-Cdt1 and mAG-Geminin intensities in the post-mitotic times further suggesting a separation between the dividing and non-dividing cells. This observation provides additional information on the increasing heterogeneity after cellular division.

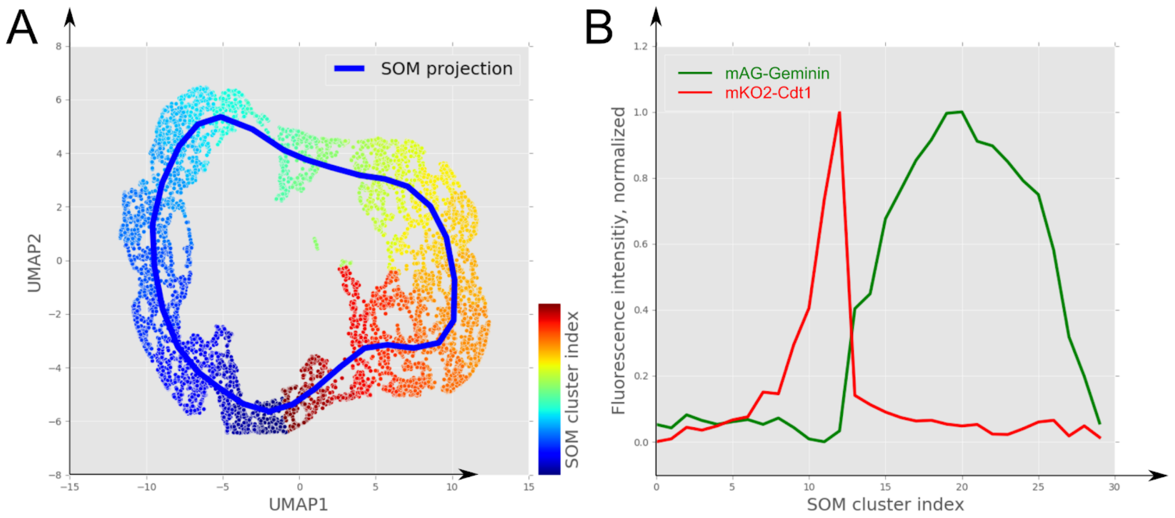

Appendix Figure S4: Initiation of the DeepCycle trajectory. To quantitatively characterize the progression of the fluorescence intensities along the UMAP projection, the neighborhood graph based SOM algorithm was employed to define the path which captures the main circular axis of this projection and was called DeepCycle trajectory. **A**. The DeepCycle trajectory (blue line) is a trajectory derived by the SOM algorithm from the UMAP coordinates. **B.** Average FUCCI2 intensities (mKO2-Cdt1: red, mAG-Geminin: green) as a progression over the SOM clusters of the training set (average n=325, total n=9753). The FUCCI trends in function of the DeepCycle pseudotime of all cells is shown in Figure 2B.

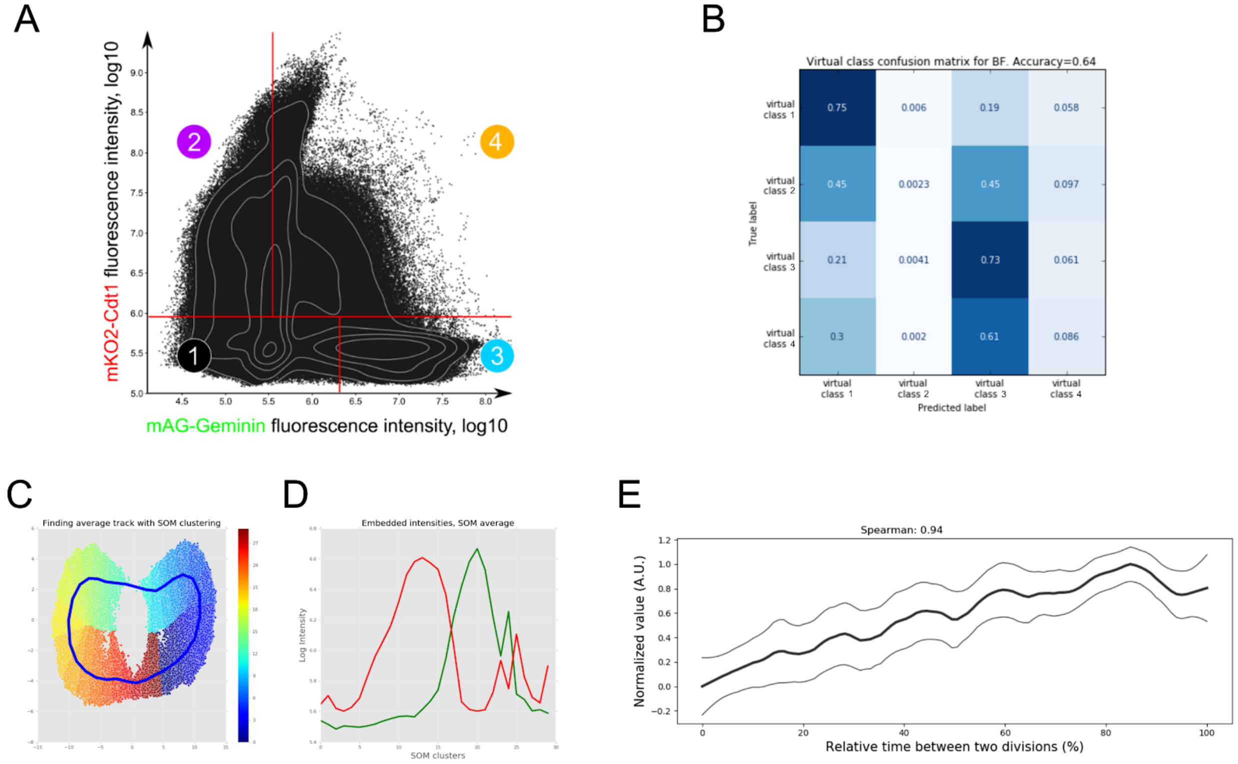

Appendix Figure S5: **Results from a DeepCycle model trained on single brighfield channel cell images. A**. Representation of the four virtual classes from the FUCCI 2 marker intensities used for the network training. This figure is a replica of Figure 1C, added here for completeness. **B**. Confusion matrix and virtual class prediction accuracies. The confusion matrix was calculated on test data. This figure is a replica of Appendix Figure S2B, added here for completeness. **C**. Low dimensional UMAP projection with the inferred DeepCycle trajectory (SOM, see Methods for more details). The colorbar indicates the SOM clusters index. **D**. Projection of the FUCCI 2 fluorescence intensities (red: mKO2-Cdt1, green: mAG-Geminin), in function of the DeepCycle pseudotime (SOM cluster index). **E**. Evaluation of the model performance at estimating the CC time quantified as the correlation between the DeepCycle pseudotime and the CC time (bold central line shows the mean, thin lines show the standard deviation, spearman r=0.94, two-sided p-value<0.001, ***, n=50). The correlation is computed for 50 cells undergoing a full cell cycle, unseen during the network training.

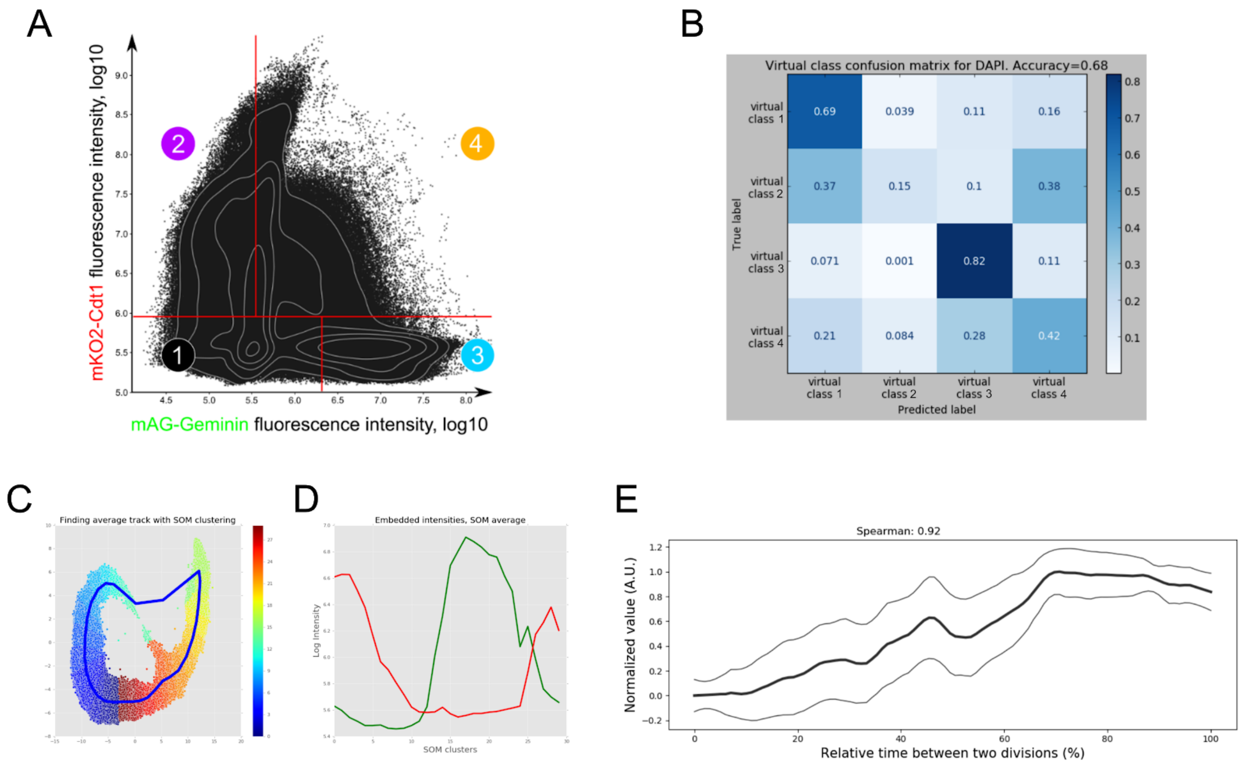

Appendix Figure S6: **Results from a DeepCycle model trained on single Hoechst channel cell images. A**. Representation of the four virtual classes from the FUCCI 2 marker intensities used for the network training. This figure is a replica of Figure 1C, added here for completeness. **B**. Confusion matrix and virtual class prediction accuracies. The confusion matrix was calculated on test data. This figure is a replica of Appendix Figure S2C, added here for completeness. **C**. Low dimensional UMAP projection with the inferred DeepCycle trajectory (SOM, see Methods for more details). The colorbar indicates the SOM clusters index. **D**. Projection of the FUCCI 2 fluorescence intensities (red: mKO2-Cdt1, green: mAG-Geminin), in function of the DeepCycle pseudotime (SOM cluster index). **E**. Evaluation of the model performance at estimating the CC time quantified as the correlation between the DeepCycle pseudotime and the CC time (bold central line shows the mean, thin lines show the standard deviation, spearman r=0.92, two-sided p-value<0.001, ***, n=50). The correlation is computed for 50 cells undergoing a full cell cycle, unseen during the network training.

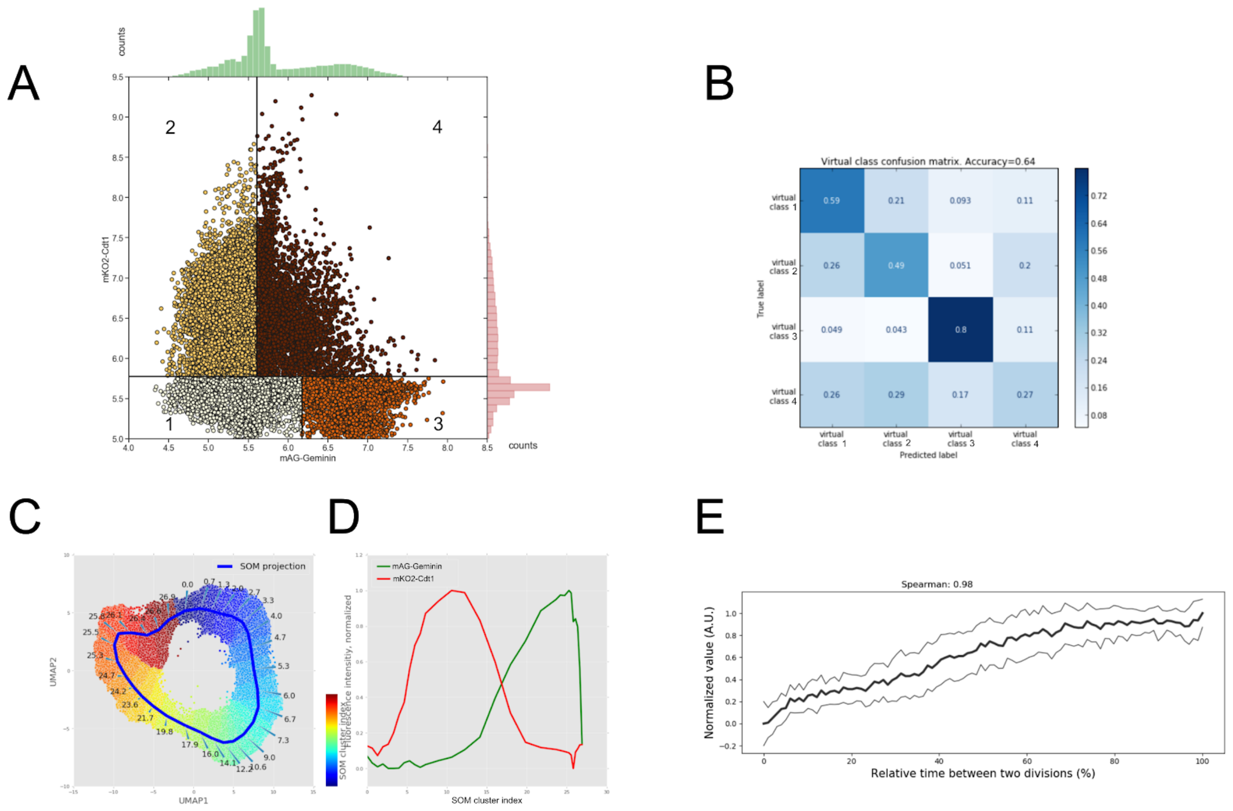

Appendix Figure S7: **Results from a DeepCycle model trained on two channel images (Hoechst and brightfield) with FUCCI2 intensities obtained from segmented nuclei. A**. Representation of the four virtual classes from the FUCCI 2 marker intensities averaged over the segmented cell nuclei. **B**. Confusion matrix and virtual class prediction accuracies. The confusion matrix was calculated on test data. **C**. Low dimensional UMAP projection with the inferred DeepCycle trajectory (SOM, see Methods for details). The jet colormap indicates the SOM cluster indexes. **D**. Projection of the FUCCI 2 fluorescence intensities (red: mKO2-Cdt1, green: mAG-Geminin), in function of the DeepCycle pseudotime (SOM cluster index). **E**. Evaluation of the model performance at estimating the CC time quantified as the correlation between the DeepCycle pseudotime and the CC time (bold central line shows the mean, thin lines show the standard deviation, spearman r=0.96, two-sided p-value<0.001, ***, n=50). The correlation is computed for 50 cells undergoing a full cell cycle, unseen during the network training.

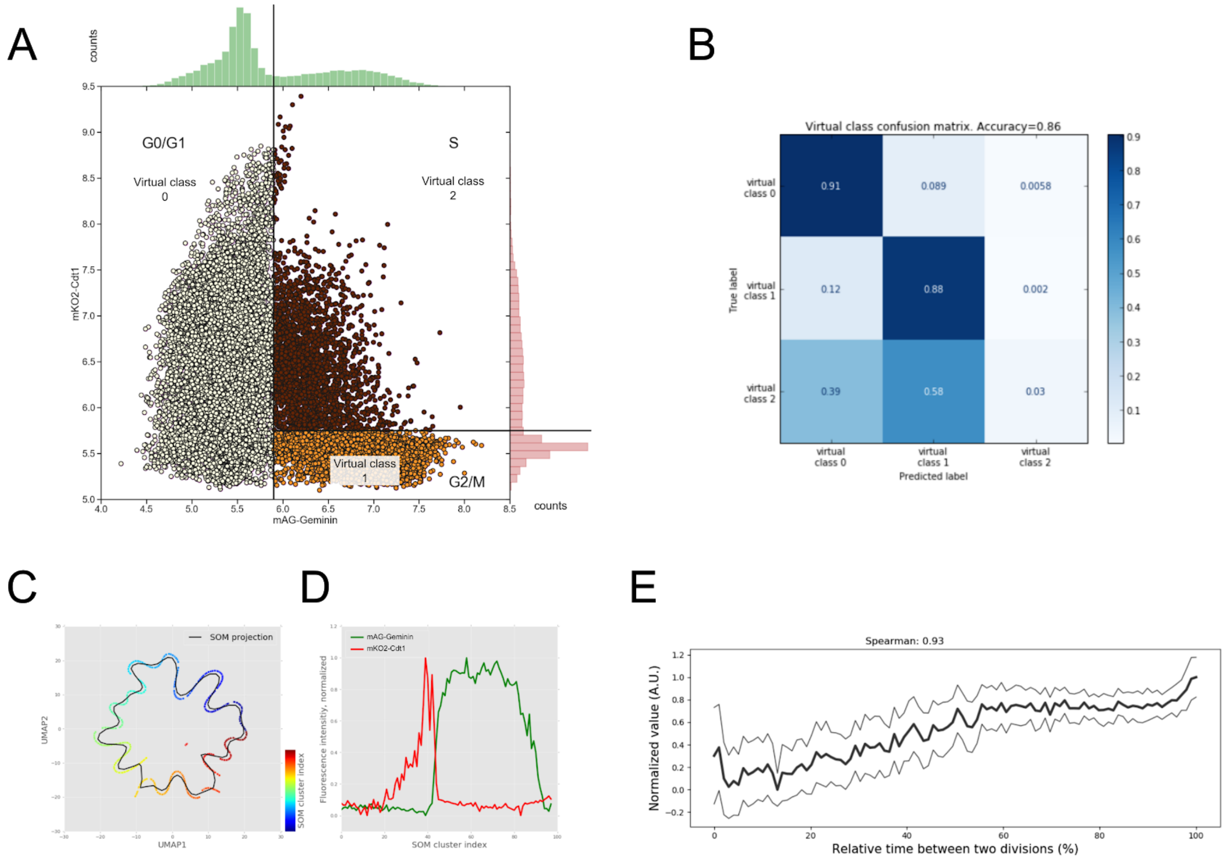

Appendix Figure S8. **Results from a DeepCycle model trained on 3 manually labelled classes approximating the cell cycle phases. A**. Representation of the three classes from the FUCCI 2 marker intensities approximating the cell cycle phases (virtual class 0, 1, 2 approximate the phases G0/G1, G2/M and S, respectively). **B**. Confusion matrix and class prediction accuracies. The confusion matrix was calculated on test data. **C**. Low dimensional UMAP projection with the inferred DeepCycle trajectory (SOM, see Methods for more details). The colorbar indicates the SOM clusters index. **D**. Projection of the FUCCI 2 fluorescence intensities (red: mKO2-Cdt1, green: mAG-Geminin), in function of the DeepCycle pseudotime (SOM cluster index). **E**. Evaluation of the model performance at estimating the CC time quantified as the correlation between the DeepCycle pseudotime and the CC time (bold central line shows the mean, thin lines show the standard deviation, spearman r=0.93, two-sided p-value<0.001, ***, n=50). The correlation is computed for 50 cells undergoing a full cell cycle, unseen during the network training.
